# 2-Amino-*N*-(2-methoxy­phen­yl)-4,5-dimethyl­thio­phene-3-carboxamide

**DOI:** 10.1107/S160053680801828X

**Published:** 2008-06-21

**Authors:** K. Chandra Kumar, M. K. Kokila, Puttaraja, J. Saravanan, Manohar V. Kulkarni

**Affiliations:** a Department of Engineering Physics, HKBK College of Engineering, Nagawara, Bangalore 560 045, Karnataka, India; b Department of Physics, Bangalore University, Bangalore 560 056, Karnataka, India; c PES College of Pharmacy, Hanumanthanagar, Bangalore 560 050, Karnataka, India; d Department of Chemistry, Karnatak University, Dharwad 580 003, Karnataka, India

## Abstract

In the title compound, C_14_H_16_N_2_O_2_S, the two aromatic rings make a dihedral angle of 13.9 (1)°. The crystal structure is stabilized by both inter- and intra­molecular N—H⋯O, C—H⋯O and C—H⋯N hydrogen bonds.

## Related literature

For related literature, see: Gewald *et al.* (1966 ▶); Cohen *et al.* (1977 ▶); Csaszar & Morvay (1983 ▶); Lakshmi *et al.* (1985 ▶); Mohan & Saravanan (2003 ▶); Bruns *et al.* (1990 ▶).
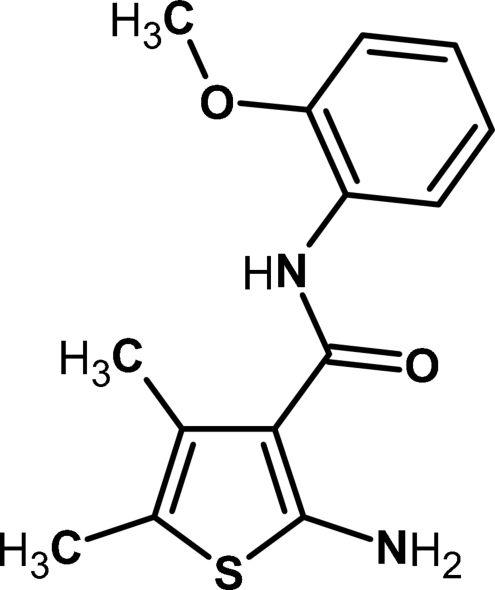


## Experimental

### 

#### Crystal data

C_14_H_16_N_2_O_2_S
*M* *_r_* = 276.35
Monoclinic, 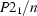

*a* = 8.606 (2) Å
*b* = 7.5193 (19) Å
*c* = 21.297 (5) Å
β = 100.599 (5)°
*V* = 1354.7 (6) Å^3^
*Z* = 4
Mo *K*α radiation
μ = 0.24 mm^−1^
*T* = 291 (2) K
0.45 × 0.35 × 0.28 mm

#### Data collection

Bruker SMART CCD area-detector diffractometer
Absorption correction: multi-scan (*SADABS*; Sheldrick, 1996 ▶) *T*_min_ = 0.908, *T*_max_ = 0.937
9834 measured reflections
2514 independent reflections
1503 reflections with *I* > 2σ(*I*)
*R*_int_ = 0.051

#### Refinement

*R*[*F* ^2^ > 2σ(*F* ^2^)] = 0.053
*wR*(*F* ^2^) = 0.142
*S* = 0.99
2514 reflections
175 parameters
H-atom parameters constrained
Δρ_max_ = 0.25 e Å^−3^
Δρ_min_ = −0.18 e Å^−3^

### 

Data collection: *SMART* (Bruker, 1998 ▶); cell refinement: *SMART*; data reduction: *SAINT* (Bruker, 1998 ▶); program(s) used to solve structure: *SHELXS97* (Sheldrick, 2008 ▶); program(s) used to refine structure: *SHELXL97* (Sheldrick, 2008 ▶); molecular graphics: *ORTEP-3 for Windows* (Farrugia, 1997 ▶); software used to prepare material for publication: *PARST* (Nardelli, 1995 ▶) and *PLATON* (Spek, 2003 ▶).

## Supplementary Material

Crystal structure: contains datablocks global, I. DOI: 10.1107/S160053680801828X/bt2721sup1.cif

Structure factors: contains datablocks I. DOI: 10.1107/S160053680801828X/bt2721Isup2.hkl

Additional supplementary materials:  crystallographic information; 3D view; checkCIF report

## Figures and Tables

**Table 1:** Hydrogen-bond geometry (Å, °)

*D*—H⋯*A*	*D*—H	H⋯*A*	*D*⋯*A*	*D*—H⋯*A*
N1—H1*A*⋯O1	0.86	2.15	2.724 (3)	124
N1—H1*B*⋯O1^i^	0.86	2.23	3.009 (4)	151
N2—H2⋯O2	0.86	2.15	2.565 (3)	109
C8—H8⋯O1	0.93	2.30	2.874 (4)	119

Symmetry code: (i) 
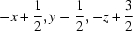
.

**Fig. 1.**
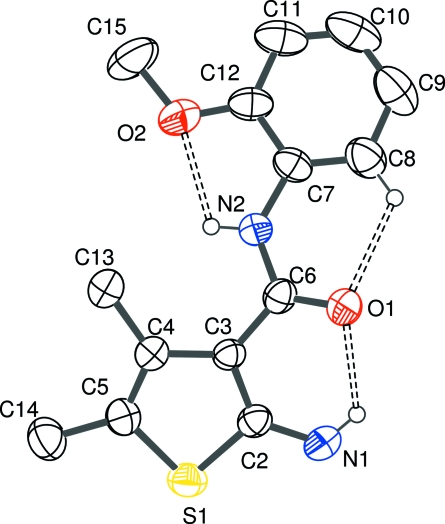
The molecular structure of (I), with atom labels and 50% probability displacement ellipsoids for non-H atoms.Dashed lines indicate intramolecular hydrogen bonds; H atoms not involved in hydrogen bonding have been omitted.

**Fig. 2.**
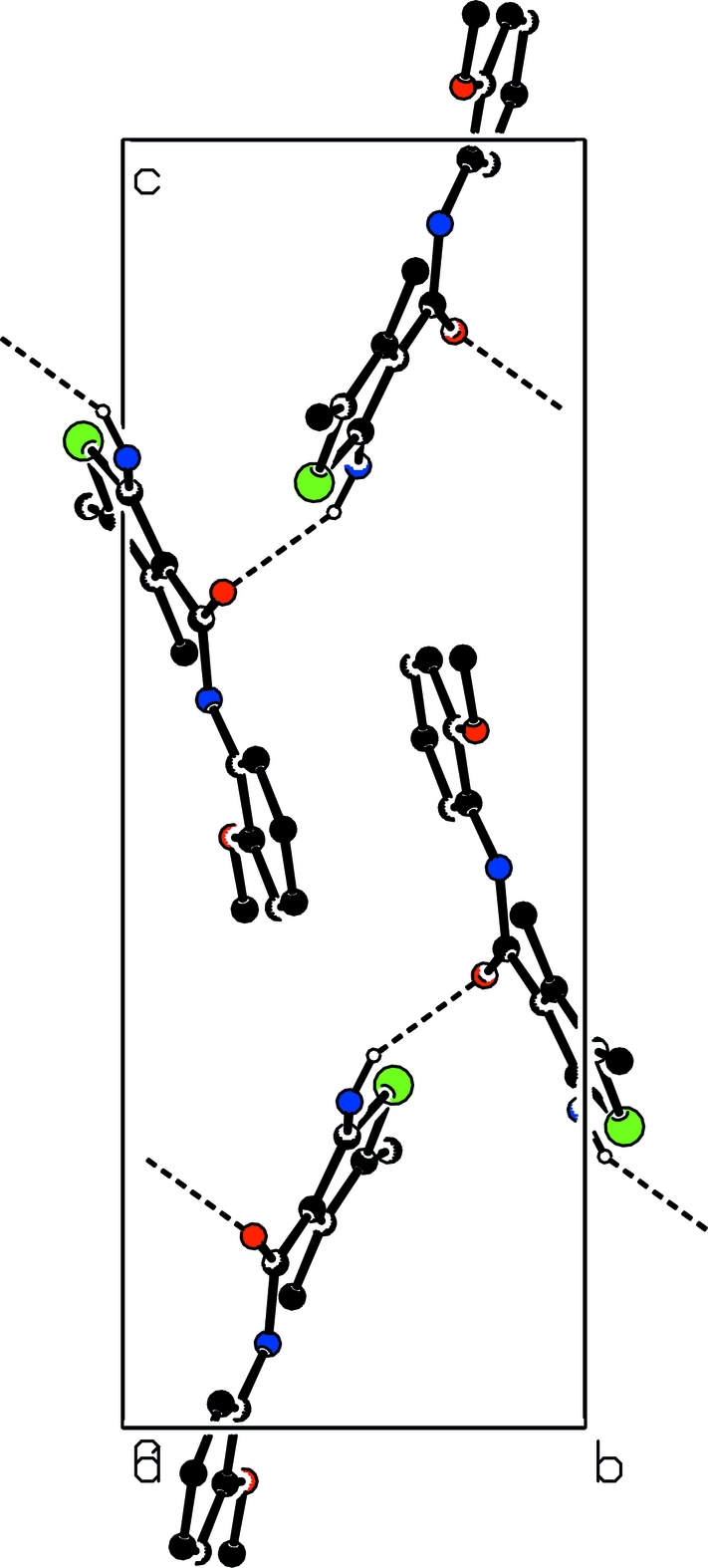
The packing of (I), viewed down the a axis shows molecules connected by N—H···O hydrogen bonds (dashedlines). H atoms not involved in hydrogen bonding have been omitted.

## supplementary crystallographic information

### Comment

Thiophene derivates containing amino and carboxyl functions have been found to exhibit anti-viral, antiinflamatory and antimicrobial activities (Mohan & Saravanan, 2003). Specifically the 2-amino-carboxylic acid esters were recognized as allosteric enhancers for A1 adenosine receptors (Bruns *et al.*, 1990).

Interaction of 3-(2-thienyl alanine) with human phenyl alanine has been studied with a view to understand the mechanism of catalysis and substrate activation. Diffraction studies on bis 5-bromo-2-substituted thiophene derivatives have revealed the existence of S—S stacking interactions. Our earlier investigations on the structures of the biologically active thiophene carboxamide, has shown that the chloro substitution in the aryl amide group had a significant effect. The *ortho*-chloro group reversed the orientation of the amide linkage and favoured the formation of more intra molecular hydrogen bonds. The *para*-chloro substitution induces stabilizing effects *via*   inter molecular hydrogen bonds. The compound in the present study bears a close structural relationship with the reported allosteric enhancers for adenosine and hence the structure has been investigated.

The molecular structure and the packing diagram  are shown in Fig. 1 and 2, respectively. The molecular structure   is stabilized by intra molecular C—H···O, N—H···O hydrogen bonds and intermolecular N—H···O interactions. (Table 2) The intra molecular C8 - H8···O1 and N1 - H1···O1 hydrogen bonds form pseudo- six membered rings and N2 - H2···O2 forms a pseudo five membered ring thus locking the molecular conformation and eliminating conformational flexibility.

### Experimental

The title compound was synthesized by mixing of ethyl methyl ketone (0.72 g, 0.01 mol) and *o*-methoxycyanoacetanilide (1.94 g, 0.01 mol) and refluxing the mixture for 1 h (Gewald *et al.*, 1966) in the presence of 4.0 ml of diethylamine. Sulfur powder (1.28 g, 0.04 mol) and 40 ml ethanol were then added and the resulting solution was heated for 2 h at 323 K. Crystals   were grown by slow evaporation in a solution of isopropyl alcohol (yield 50%).

### Refinement

H atoms were positioned geometrically [N—H = 0.86 Å, and C— H = 0.93 (CH), 0.97 (CH_2_) and 0.96 Å (CH_3_)] and constrained to ride on their parent atoms with *U*_iso_(H) values of 1.2 (1.5 for methyl) times *U*_eq_(C, N). A rotating group model was used for the methyl groups.

### Crystal data

**Table:**

C_14_H_16_N_2_O_2_S	*F*_000_ = 584
*M**_r_* = 276.35	*D*_x_ = 1.355 Mg m^−^^3^
Monoclinic, *P*2_1_/*n*	Mo *K*α radiation λ = 0.71073 Å
Hall symbol: -P2yn	Cell parameters from 670 reflections
*a* = 8.606 (2) Å	θ = 2.0–28.5º
*b* = 7.5193 (19) Å	µ = 0.24 mm^−^^1^
*c* = 21.297 (5) Å	*T* = 291 (2) K
β = 100.599 (5)º	Block, yellow
*V* = 1354.7 (6) Å^3^	0.45 × 0.35 × 0.28 mm
*Z* = 4	

### Data collection

**Table:**

Bruker SMART CCD area-detector diffractometer	2514 independent reflections
Radiation source: fine-focus sealed tube	1503 reflections with *I* > 2σ(*I*)
Monochromator: graphite	*R*_int_ = 0.051
*T* = 291(2) K	θ_max_ = 25.5º
ψ and ω scans	θ_min_ = 2.0º
Absorption correction: multi-scan(SADABS; Sheldrick, 1996)	*h* = −10→10
*T*_min_ = 0.908, *T*_max_ = 0.937	*k* = −9→9
9834 measured reflections	*l* = −25→23

### Refinement

**Table:**

Refinement on *F*^2^	Secondary atom site location: difference Fourier map
Least-squares matrix: full	Hydrogen site location: inferred from neighbouring sites
*R*[*F*^2^ > 2σ(*F*^2^)] = 0.053	H-atom parameters constrained
*wR*(*F*^2^) = 0.142	*w* = 1/[σ^2^(*F*_o_^2^) + (0.0749*P*)^2^] where *P* = (*F*_o_^2^ + 2*F*_c_^2^)/3
*S* = 0.99	(Δ/σ)_max_ = 0.001
2514 reflections	Δρ_max_ = 0.25 e Å^−^^3^
175 parameters	Δρ_min_ = −0.18 e Å^−^^3^
Primary atom site location: structure-invariant direct methods	Extinction correction: none

### Special details

**Table:**

Geometry. All e.s.d.'s (except the e.s.d. in the dihedral angle between two l.s. planes) are estimated using the full covariance matrix. The cell e.s.d.'s are taken into account individually in the estimation of e.s.d.'s in distances, angles and torsion angles; correlations between e.s.d.'s in cell parameters are only used when they are defined by crystal symmetry. An approximate (isotropic) treatment of cell e.s.d.'s is used for estimating e.s.d.'s involving l.s. planes.
Refinement. Refinement of *F*^2^ against ALL reflections. The weighted *R*-factor *wR* and goodness of fit *S* are based on *F*^2^, conventional *R*-factors *R* are based on *F*, with *F* set to zero for negative *F*^2^. The threshold expression of *F*^2^ > σ(*F*^2^) is used only for calculating *R*-factors(gt) *etc*. and is not relevant to the choice of reflections for refinement. *R*-factors based on *F*^2^ are statistically about twice as large as those based on *F*, and *R*- factors based on ALL data will be even larger.

### Fractional atomic coordinates and isotropic or equivalent isotropic displacement parameters (Å^2^)

**Table:**

	*x*	*y*	*z*	*U*_iso_*/*U*_eq_	
N1	0.2740 (3)	0.5092 (4)	0.74695 (12)	0.0593 (8)	
H1A	0.2056	0.5582	0.7662	0.071*	
H1B	0.2436	0.4570	0.7109	0.071*	
N2	0.4683 (3)	0.6863 (3)	0.93475 (11)	0.0480 (7)	
H2	0.5646	0.6512	0.9455	0.058*	
O1	0.2675 (3)	0.7169 (3)	0.85124 (9)	0.0630 (7)	
O2	0.6570 (3)	0.7367 (3)	1.04138 (10)	0.0676 (7)	
S1	0.56490 (10)	0.41424 (12)	0.73395 (4)	0.0535 (3)	
C2	0.4302 (3)	0.5144 (4)	0.77352 (13)	0.0420 (7)	
C3	0.5040 (3)	0.5902 (4)	0.83034 (12)	0.0377 (7)	
C4	0.6745 (3)	0.5669 (4)	0.84070 (13)	0.0398 (7)	
C5	0.7229 (3)	0.4775 (4)	0.79298 (15)	0.0467 (7)	
C6	0.4057 (3)	0.6700 (4)	0.87196 (14)	0.0420 (7)	
C7	0.3992 (4)	0.7521 (4)	0.98507 (14)	0.0473 (8)	
C8	0.2397 (4)	0.7879 (4)	0.98133 (16)	0.0627 (9)	
H8	0.1694	0.7726	0.9430	0.075*	
C9	0.1854 (5)	0.8470 (5)	1.0354 (2)	0.0760 (11)	
H9	0.0784	0.8705	1.0332	0.091*	
C10	0.2894 (6)	0.8706 (5)	1.09189 (19)	0.0783 (12)	
H10	0.2519	0.9096	1.1278	0.094*	
C11	0.4478 (5)	0.8375 (4)	1.09617 (16)	0.0681 (11)	
H11	0.5174	0.8549	1.1346	0.082*	
C12	0.5028 (4)	0.7782 (4)	1.04318 (14)	0.0528 (8)	
C13	0.7904 (4)	0.6333 (4)	0.89802 (14)	0.0574 (9)	
H13A	0.7849	0.5590	0.9342	0.086*	
H13B	0.7642	0.7534	0.9074	0.086*	
H13C	0.8955	0.6298	0.8889	0.086*	
C14	0.8866 (4)	0.4251 (5)	0.78467 (17)	0.0650 (9)	
H14A	0.9630	0.4970	0.8119	0.097*	
H14B	0.8970	0.4428	0.7410	0.097*	
H14C	0.9043	0.3021	0.7958	0.097*	
C15	0.7716 (5)	0.7639 (6)	1.09758 (16)	0.0876 (13)	
H15A	0.7754	0.8879	1.1086	0.131*	
H15B	0.8733	0.7265	1.0902	0.131*	
H15C	0.7435	0.6959	1.1320	0.131*	

### Atomic displacement parameters (Å^2^)

**Table:**

	*U*^11^	*U*^22^	*U*^33^	*U*^12^	*U*^13^	*U*^23^
N1	0.0488 (17)	0.082 (2)	0.0435 (15)	−0.0024 (14)	−0.0015 (13)	−0.0087 (14)
N2	0.0424 (14)	0.0643 (17)	0.0367 (14)	0.0069 (12)	0.0056 (12)	−0.0021 (12)
O1	0.0517 (14)	0.0900 (18)	0.0449 (13)	0.0223 (12)	0.0019 (11)	0.0006 (12)
O2	0.0649 (16)	0.0908 (18)	0.0437 (13)	−0.0020 (14)	0.0012 (12)	−0.0051 (12)
S1	0.0587 (6)	0.0575 (5)	0.0451 (5)	−0.0032 (4)	0.0117 (4)	−0.0088 (4)
C2	0.0434 (17)	0.0445 (16)	0.0374 (16)	−0.0041 (14)	0.0055 (14)	0.0028 (13)
C3	0.0402 (17)	0.0392 (15)	0.0327 (15)	0.0016 (13)	0.0043 (13)	0.0021 (13)
C4	0.0418 (17)	0.0356 (15)	0.0410 (17)	−0.0004 (13)	0.0051 (13)	0.0033 (13)
C5	0.0457 (18)	0.0442 (17)	0.0508 (18)	0.0008 (14)	0.0106 (15)	0.0013 (14)
C6	0.0418 (18)	0.0429 (17)	0.0395 (17)	0.0021 (14)	0.0031 (14)	0.0067 (13)
C7	0.058 (2)	0.0443 (18)	0.0433 (18)	0.0022 (15)	0.0187 (16)	0.0033 (14)
C8	0.067 (2)	0.070 (2)	0.055 (2)	0.0124 (18)	0.0222 (18)	0.0090 (18)
C9	0.083 (3)	0.075 (3)	0.081 (3)	0.024 (2)	0.044 (2)	0.020 (2)
C10	0.126 (4)	0.061 (2)	0.060 (3)	0.019 (2)	0.046 (3)	0.0082 (19)
C11	0.109 (3)	0.055 (2)	0.044 (2)	0.002 (2)	0.023 (2)	0.0019 (16)
C12	0.075 (2)	0.0466 (18)	0.0370 (18)	−0.0016 (17)	0.0123 (17)	0.0028 (14)
C13	0.0458 (19)	0.068 (2)	0.055 (2)	0.0007 (16)	0.0024 (16)	−0.0063 (17)
C14	0.056 (2)	0.066 (2)	0.075 (2)	0.0058 (18)	0.0185 (19)	−0.0091 (19)
C15	0.088 (3)	0.123 (3)	0.044 (2)	−0.026 (3)	−0.010 (2)	0.007 (2)

### Geometric parameters (Å, °)

**Table:**

N1—C2	1.360 (3)	C7—C8	1.387 (5)
N1—H1A	0.8600	C8—C9	1.392 (5)
N1—H1B	0.8600	C8—H8	0.9300
N2—C6	1.352 (3)	C9—C10	1.373 (5)
N2—C7	1.407 (4)	C9—H9	0.9300
N2—H2	0.8600	C10—C11	1.373 (5)
O1—C6	1.241 (3)	C10—H10	0.9300
O2—C12	1.370 (4)	C11—C12	1.376 (4)
O2—C15	1.419 (4)	C11—H11	0.9300
S1—C2	1.727 (3)	C13—H13A	0.9600
S1—C5	1.740 (3)	C13—H13B	0.9600
C2—C3	1.382 (4)	C13—H13C	0.9600
C3—C4	1.454 (4)	C14—H14A	0.9600
C3—C6	1.462 (4)	C14—H14B	0.9600
C4—C5	1.347 (4)	C14—H14C	0.9600
C4—C13	1.512 (4)	C15—H15A	0.9600
C5—C14	1.504 (4)	C15—H15B	0.9600
C7—C12	1.399 (4)	C15—H15C	0.9600
			
C2—N1—H1A	120.0	C8—C9—H9	119.9
C2—N1—H1B	120.0	C10—C9—H9	119.9
H1A—N1—H1B	120.0	C11—C10—C9	120.9 (4)
C6—N2—C7	129.6 (3)	C11—C10—H10	119.5
C6—N2—H2	115.2	C9—C10—H10	119.5
C7—N2—H2	115.2	C10—C11—C12	119.5 (4)
C12—O2—C15	118.0 (3)	C10—C11—H11	120.3
C2—S1—C5	91.95 (14)	C12—C11—H11	120.3
N1—C2—C3	129.5 (3)	O2—C12—C11	125.3 (3)
N1—C2—S1	119.1 (2)	O2—C12—C7	114.0 (3)
C3—C2—S1	111.4 (2)	C11—C12—C7	120.8 (3)
C2—C3—C4	111.8 (3)	C4—C13—H13A	109.5
C2—C3—C6	118.4 (2)	C4—C13—H13B	109.5
C4—C3—C6	129.6 (2)	H13A—C13—H13B	109.5
C5—C4—C3	112.9 (3)	C4—C13—H13C	109.5
C5—C4—C13	121.6 (3)	H13A—C13—H13C	109.5
C3—C4—C13	125.4 (3)	H13B—C13—H13C	109.5
C4—C5—C14	130.2 (3)	C5—C14—H14A	109.5
C4—C5—S1	111.9 (2)	C5—C14—H14B	109.5
C14—C5—S1	117.9 (2)	H14A—C14—H14B	109.5
O1—C6—N2	120.5 (3)	C5—C14—H14C	109.5
O1—C6—C3	121.6 (3)	H14A—C14—H14C	109.5
N2—C6—C3	117.9 (2)	H14B—C14—H14C	109.5
C12—C7—N2	115.7 (3)	O2—C15—H15A	109.5
C12—C7—C8	119.1 (3)	O2—C15—H15B	109.5
N2—C7—C8	125.2 (3)	H15A—C15—H15B	109.5
C9—C8—C7	119.6 (4)	O2—C15—H15C	109.5
C9—C8—H8	120.2	H15A—C15—H15C	109.5
C7—C8—H8	120.2	H15B—C15—H15C	109.5
C8—C9—C10	120.2 (4)		
			
C5—S1—C2—N1	179.1 (2)	C4—C3—C6—O1	163.3 (3)
C5—S1—C2—C3	−1.1 (2)	C2—C3—C6—N2	157.1 (3)
N1—C2—C3—C4	−179.5 (3)	C4—C3—C6—N2	−18.8 (4)
S1—C2—C3—C4	0.8 (3)	C6—N2—C7—C12	−170.1 (3)
N1—C2—C3—C6	3.9 (5)	C6—N2—C7—C8	11.3 (5)
S1—C2—C3—C6	−175.8 (2)	C12—C7—C8—C9	−0.7 (5)
C2—C3—C4—C5	0.1 (3)	N2—C7—C8—C9	177.8 (3)
C6—C3—C4—C5	176.3 (3)	C7—C8—C9—C10	0.4 (5)
C2—C3—C4—C13	−179.9 (3)	C8—C9—C10—C11	0.3 (6)
C6—C3—C4—C13	−3.8 (5)	C9—C10—C11—C12	−0.6 (5)
C3—C4—C5—C14	−179.9 (3)	C15—O2—C12—C11	−3.0 (5)
C13—C4—C5—C14	0.1 (5)	C15—O2—C12—C7	178.2 (3)
C3—C4—C5—S1	−1.0 (3)	C10—C11—C12—O2	−178.6 (3)
C13—C4—C5—S1	179.1 (2)	C10—C11—C12—C7	0.3 (5)
C2—S1—C5—C4	1.2 (2)	N2—C7—C12—O2	0.7 (4)
C2—S1—C5—C14	−179.7 (3)	C8—C7—C12—O2	179.4 (3)
C7—N2—C6—O1	0.1 (5)	N2—C7—C12—C11	−178.3 (3)
C7—N2—C6—C3	−177.8 (3)	C8—C7—C12—C11	0.4 (5)
C2—C3—C6—O1	−20.8 (4)		

### Hydrogen-bond geometry (Å, °)

**Table:**

*D*—H···*A*	*D*—H	H···*A*	*D*···*A*	*D*—H···*A*
N1—H1A···O1	0.86	2.15	2.724 (3)	124
N1—H1B···O1^i^	0.86	2.23	3.009 (4)	151
N2—H2···O2	0.86	2.15	2.565 (3)	109
C8—H8···O1	0.93	2.30	2.874 (4)	119

Symmetry codes: (i) −*x*+1/2, *y*−1/2, −*z*+3/2.
